# The Lipoxygenase Gene Family in Poplar: Identification, Classification, and Expression in Response to MeJA Treatment

**DOI:** 10.1371/journal.pone.0125526

**Published:** 2015-04-30

**Authors:** Zhu Chen, Xue Chen, Hanwei Yan, Weiwei Li, Yuan Li, Ronghao Cai, Yan Xiang

**Affiliations:** 1 Laboratory of Modern Biotechnology, Anhui Agricultural University, Hefei, China; 2 Key Laboratory of Biomass Improvement and Conversion, Anhui Agriculture University, Hefei, China; East Carolina University, UNITED STATES

## Abstract

**Background:**

Lipoxygenases (LOXs) are important dioxygenases in cellular organisms. LOXs contribute to plant developmental processes and environmental responses. However, a systematic and comprehensive analysis has not been focused on the *LOX* gene family in poplar. Therefore, in the present study, we performed a comprehensive analysis of the *LOX* gene family in poplar.

**Results:**

Using bioinformatics methods, we identified a total of 20 *LOX* genes. These *LOX* genes were clustered into two subfamilies. The gene structure and motif composition of each subfamily were relatively conserved. These genes are distributed unevenly across nine chromosomes. The *PtLOX* gene family appears to have expanded due to high tandem and low segmental duplication events. Microarray analysis showed that a number of *PtLOX* genes have different expression pattern across disparate tissues and under various stress treatments. Quantitative real-time PCR (qRT-PCR) analysis was further performed to confirm the responses to MeJA treatment of the 20 poplar *LOX* genes. The results show that the *PtLOX* genes are regulated by MeJA (Methyl jasmonate) treatment.

**Conclusions:**

This study provides a systematic analysis of *LOX* genes in poplar. The gene family analysis reported here will be useful for conducting future functional genomics studies to uncover the roles of *LOX* genes in poplar growth and development.

## Introduction

As an important tree species of shelterbelt and timber forest in our country, poplar trees have enormous economic and ecological benefits, as well as unique biological properties of basic scientific interest [1, 2]. However, poplar trees are affected by a variety of environmental stresses, such as the rapid spread of pests, which has led to tremendous economic loss [3, 4]. Increasing tolerance to pests and insects in order to improve both the quality and quantity of available poplar wood is one of the major aims of researchers working in this area [5].

In plants, some oxylipins [6, 7] and their derivatives, such as jasmonic acid (JA) [8], leaf aldehydes [9, 10] and divinyl ethers [11], have been suggested to be involved in plant defense against pests. Biosynthetic pathways that lead to the formation of these oxylipins in plants have been investigated. Such compounds are produced via the lipoxygenase (LOX) pathway [12].

LOX proteins (EC 1.13.11.12) are monomeric, nonheme iron that contain dioxygenases; they are widely expressed in animals, plants and fungi [13]. Polysaturated fatty acids (PUFA) that contain a *cis*, *cis*-1,4-pentadiene structural unit, such as linoleic acid (LA), α-linolenic acid and arachidonic acid, are LOX substrates. Plant LOXs are generally classified according to the specificity of their action on the substrate [14]. This substrate is oxygenated at either the C-atom 9 or C-atom 13 of the fatty acid hydrocarbon backbone. Thus, plant LOXs have been divided into two classes, 9-LOXs and 13-LOXs. According to their primary structure and overall sequence similarity, plant LOXs can be grouped into two subfamilies. Type I LOX proteins harbor no transit peptides and exhibit high sequence similarity (>75%). By contrast, type II LOX proteins carry a transit peptide sequence and show only moderate overall sequence similarity (~35%). To date, these LOX forms all belong to the subfamily of 13-LOXs [15, 16].

LOX proteins and LOX-derived products are involved in a series of biological events, such as seed germination [17], tuber development [18], sex determination [19], fruit ripening [20], and plant defense responses [21, 22]. LOXs are widely known to oxygenase polyunsaturated fatty acids (PUFAs) to produce either 13- or 9-fatty acid hydroperoxides. Hydroperoxides are then rapidly converted into diverse oxylipins [23]. In plants, oxylipins contribute to insect resistance mechanisms through two pathways. Firstly, oxylipins such as jasmonic acid (JA) that are associated with plant defenses are subsequently formed from the octadecanoid pathway via the LOX-mediated oxidation of PUFA at the 13-carbon site [8, 24]. Secondly, oxylipins such as the C6 volatile are widely known as green leaf volatiles (GLVs) because of their characteristic aroma, which is often associated with the scent of freshly-cut grass. It has been observed that different forms of GLVs play an important role in plant resistance [9, 10, 24].

Extensive research has detected the resistance of pests to LOX in a wide range of plants. In *Arabidopsis thaliana*, the expression of *AtLOX1* is stimulated by stress-related hormones and *AtLOX2* has been identified as specifically involved in jasmonate biosynthesis [25]. Previous studies have indicated that *TomloxD* is rapidly and transiently induced by damage in tomato [26, 27]. Antisense approaches in potato have shown that the expression of *LoxH1* is detectable and involved in the generation of volatile defense systems [28]. In maize, the activity of *ZmLOX3* is required for normal levels of resistance to root-knot nematodes [29]. *AdLOX3*, *AdLOX4* and *AdLOX6* are candidates for the regulation of the synthesis of kiwifruit volatile compounds [30]. *VvLOXO* plays a part in the response of grape berries to environmental and stress-related stimuli [31]. Recent evidence from literature reviews has shown that *MdLOX5* genes might be responsible for aphid tolerance or resistance [32].

Previous work on LOX activity and the expression of two members of the *LOX* gene family in poplar has suggested that LOXs function in stress tolerance in trees [25]. This suggested role for LOX in poplar has prompted a more detailed analysis of the poplar *LOX* gene family. However, no genome-wide characterization of the *LOX* gene family has been performed in poplar to date.

In this study, 20 putative *LOX* genes were identified and characterized in the poplar genome. The responses to MeJA treatment of all of these genes were investigated using qRT-PCR analyses. Our preliminary results provide insights that will be useful for the further investigation of the roles of these candidate genes in poplar stress responses.

## Materials and Methods

### Ethics statement

No specific permits were required for the described field studies. No specific permissions were required for these locations and activities. The location is not privately-owned or protected in any way and the field studies did not involve endangered or protected species.

### Database search and sequence retrieval

Sequences of Arabidopsis LOX proteins were obtained from the Arabidopsis Information Resource (TAIR, http://www.Arabidopsis.org/, release 10.0). Sequences of *Cucumis sativus*, *Glycine max*, *Hordeum vulgare*, *Lens culinaris*, *Lycopersicon esculentum*, *Nicotiana attenuata*, *Nicotiana tabacum*, *Oryza sativa*, *Pisum sativum*, *Phaseolus vulgaris*, *Solanum tuberosum*, *Zea mays*, *Vitis vinifera*, *Actindia deliciosa*, and *Triticum aestivum* were downloaded from NCBI (http://www.ncbi.nlm.nih.gov/). Sequences of Populus were downloaded from Phytozome (http://www.phytozome.net/). Local Blast searching was performed using Arabidopsis LOX proteins as queries for the identification of *LOX* genes from poplar. For the misannotated genes, manual reannotation was performed using the online web server Pfam (http://pfam.sanger.ac.uk/)[26]. All of the sequences were further manually analyzed to confirm the presence of LOX domain and PLAT/LH2 (polycystin-1, lipoxygenase, α-toxin domain or the lipoxygenase homology) domain using the InterProScan program (http://www.ebi.ac.uk/Tools/InterProScan/) [27].

### Phylogenetic analysis

Eighty-four plant lipoxygenase amino acid sequences were analyzed, including twenty PtLOXs. Conserved protein regions were aligned using ClustalX 2.0 software [28] and adjusted manually with BioEdit [29]. NJ trees were generated using MEGA 5.0 [30], with 1000 bootstrap replicates. MrBayes software [31] was used to construct BI trees, using the WAG model of evolution, after running for 1,000,000 generations, with four Markov chains sampled every 1000 generations.

### Gene structure analysis and identification of conserved motifs

The exon/intron organization of *LOX* genes was generated online using Gene structure display server (GSDS; http://gsds.cbi.pku.edu.cn/) by alignment of the cDNAs with their corresponding genomic DNA sequences [32]. Structural motif annotation was performed using the MEME program [33]. The maximum number of motifs was set at 20, and the optimum motif widths were set at between six and 200 residues. Structural motif annotation was performed using the Pfam program. Subcellular localization was predicted using the TargetP 1.1 (http://www.cbs.dtu.dk/services/TargetP/) server, ProtComp 9.0 (http://linux1.softberry.com/berry.phtml), Cell-PLoc (http://www.csbio.sjtu.edu.cn/bioinf/Cell-PLoc/) and WoLF PSORT program (http://wolfpsort.org/) [34].

### Chromosomal location and gene duplication

Information about the chromosome locations of the genes was obtained from the Phytozome database. The distribution of *PtLOX* gene family members throughout the poplar genome was generated using MapInspect software. All these genes were compared by pairwise BLASTP (E-value<10^-10^) analysis [35]. Data from the recently identified duplicated blocks were obtained [36], and the segment duplication coordinates of the target genes were obtained from the Poplar JGI browser. Genes separated by five or fewer gene loci in a 60-kb region were considered to be tandem duplicates.

### Calculation of Ka/Ks values

Amino acid sequences from segmentally duplicated pairs were first aligned with Clustal 2.0, and the aligned sequences were subsequently transferred into original cDNA sequences using the PAL2NAL program (http://www.bork.embl.de/pal2nal/) [37], which uses the CODEML program of PAML to estimate synonymous (Ks) and nonsynonymous (Ka) substitution rates. Divergence time (T) was calculated using a synonymous mutation rate of one substitution per synonymous site per year as T = Ks/2 λ (λ = 9.161029 for Populus) [38].

### Microarray analysis

A record of gene expression in tissues was obtained from the Poplar eFP Browser (http://bar.utoronto.ca/efppop/cgi-bin/efpWeb.cgi)[39]. The genome-wide microarray data were obtained from the Gene Expression Omnibus database at the National Center for Biotechnology Information under series accession numbers GSE13990, GSE16786 and GSE23726. The data were then imported into Cluster (version3.0) to generate heatmaps. Probe sets corresponding to the putative poplar *LOX* genes were identified using an online Probe Match tool available at the NetAffx Analysis Center (http://www.affymetrix.com/). For genes with more than one probe sets, the median of expression values were considered. When several genes have the same probe set, then they are considered to have same level of transcript abundance [40–43].

### Plant material collection

Young leaves were harvested of 1-year-old Nanlin-95 (Populus× euramericana cv. ‘Nanlin95’) plants that were grown in a greenhouse (16 h light/8 h dark, 25°C–28°C). MeJA stress treatment was conducted following a previously published method with minor modifications [25], the amount was controlled such that all leaves were densely covered with the suspension. MeJA (Sigma, 95%) was diluted 1:10 with 95% ethanol, followed by a further dilution with MilliQ water containing 0.1% Triton X-100, resulting in a final concentration of 100 μmol/L MeJA. As above, prior to each treatment, leaves were harvested for the controls. Three replicates from three independent plants were collected. Treated leaves were then harvested at 0.5, 1, 3, 6, 9, 12 and 24 h post-treatment, frozen quickly in liquid nitrogen, and stored at -70°C.

### RNA extraction and qRT-PCR analysis

Total RNA from young leaves was extracted using TRIzol reagent (Shenggong, Shanghai, China) according to the manufacturer’s instructions. RNA integrity was verified by 2% agar gel electrophoresis. RNA was treated with Recombinant DNase I (RNase-free) (TaKaRa) to romove potentially contaminating genomic DNA. The first-strand cDNA was then synthesized using a PrimerScriptTM RT Reagent Kit (TaKaRa). Primers were designed using Primer5.0, and their specificity was checked using information obtained from the NCBI website (S1 Table). Subsequently, by performing analysis of melting curves and analysis of visualization of amplicon fragments, we found primers were gene-specific only when corresponding melting curves generated a single sharp peak and the primers demonstrated an electrophoresis pattern of a single amplicon with the correct predicted length. The qRT-PCR analysis was conducted using 2×SYBR Premix Ex Taq (TaKaRa). The reactions were prepared in a total volume of 25 μl containing 3 μl of template, 12.5 μl of 2×SYBR Premix, 1 μl of each specific primer (10 μM), and 7.5 μl ddH2O. The reactions were performed under the following conditions: 95°C for 30 s, 40 cycles of 5 s at 95°C, and 55°C for 34s. Every treatment included three replicates, and each reaction was run in triplicate. Relative gene expression with respect to the internal reference gene UBQ10 was determined as described previously [40–42, 44]. Standard curve analysis of all the primer pairs were performed (S1 Fig). The information of the primers and amplification efficiency of all the primer pairs were list in S1 Table. The relative mRNA level for each gene was calculated as 2^-ΔΔCT^ values in comparison to that of unstressed leaves[45]. The relative expression levels were calculated as 2^-ΔΔCT^ [ΔCT = CT, _Target_—CT, _UBQ10_. ΔΔCT = ΔCT, treatment- ΔCT, _CK (0 h)_]. The relative expression levels (2^-ΔΔCT^, _CK (0 h)_) in the untreated control plants were normalized to 1 as described previously [45–47]. If an efficiency of amplification was less than 2, the result was proofread. Statistical analyses were conducted using GraphPad Prism 5.01 software [48].

## Results and Discussion

### Identification of *LOX* gene family in poplar

A comprehensive genome-wide search of the *Populus* database using the AtLOX protein sequences resulted in the identification of 20 *LOX* genes [49]. All of the deduced proteins had features of the plant *LOX* gene family as described previously [15]. We excluded several genes that encoded for proteins containing only one lipoxygenase domain [50, 51] or a PLAT/LH2 domain [52, 53].

The number of *LOX* genes in *Populus trichocarpa* is approximately three-fold that of Arabidopsis [49], which is not consistent with the ratio of 1.4–1.6 putative poplar homologs per Arabidopsis gene that was predicted from comparative genomics studies. According to the present study, the number of Populus *LOX* genes was greater than that of Arabidopsis, which was also the case for the cucumber [54], grape [55], and apple [56] *LOX* gene families. The poplar *LOX* genes were designated *PtLOXs* according to nomenclature proposed in a previous study.

Detailed information about the *LOX* family of genes in *Populus*, including accession numbers and similarities to their Arabidopsis orthologs, is listed in Table 1. The length of PtLOX proteins was constant at between 796 and 927 amino acids, and the predicted open reading frames ranged from 2,391 to 2,784 bp. The calculated molecular masses of the 20 *PtLOXs* ranged from 90.9 to 105.6 kDa, with *PtLOX14* being the largest. Sequence comparison revealed that the genes shared high levels of sequence homology at the nucleotide level (44.1% to 99.4% similartiy) within the coding region and at the amino acid level (39.9% to 99.3% similartiy) (S2 Table). Variations in the PI values of PtLOX proteins indicated that individual PtLOX proteins may require different ionic strengths, pH, and the presence of a reducing agent for their optimal performance.

**Table 1:** Detailed information about the LOX gene family in Populus.

Name	Locus name	Location coordinates	Protein			Chr.	ORF length	Arabidopsis ortholog locus
		(5'–3')	Length (a.a.)	Mol.Wt. (Da)	PI		(bp)	
*PtLOX1*	Potri.001G015300	1076313–1081197	898	102243	7.01	1	2697	AT3G45140
*PtLOX2*	Potri.001G015400	1090420–1098069	902	102293.9	6.29	1	2709	
*PtLOX3*	Potri.001G015500	1105670–1110895	898	102645.8	5.86	1	2697	
*PtLOX4*	Potri.001G015600	1118168–1123930	898	102654.8	5.71	1	2697	
*PtLOX5*	Potri.001G167700	14106872–14112847	923	104071	8.12	1	2772	AT1G17420AT1G67560
*PtLOX6*	Potri.003G067600	9576888–9583048	925	104519.6	7.37	3	2778	AT1G17420AT1G67561
*PtLOX7*	Potri.005G032400	2425802–2431106	866	98665.9	5.47	5	2601	
*PtLOX8*	Potri.005G032600	2435033–2439658	796	90918.2	5.48	5	2391	
*PtLOX9*	Potri.005G032700	2451619–2456194	866	98659.7	5.34	5	2601	
*PtLOX10*	Potri.005G032800	2462946–2469256	863	98546	5.51	5	2592	
*PtLOX11*	Potri.008G151500	10276751–10281394	880	100532.8	6.04	8	2643	AT3G22400
*PtLOX12*	Potri.008G178000	12146645–12151320	927	105043.9	6.63	8	2784	AT1G72520
*PtLOX13*	Potri.009G022400	3421114–3425183	901	102121.7	6.47	9	2706	
*PtLOX14*	Potri.010G057100	8651258–8655916	926	105641.2	6.63	10	2781	
*PtLOX15*	Potri.010G089500	11305668–11310367	881	100751	6.46	10	2646	AT3G22400
*PtLOX16*	Potri.013G022000	1454479–1459136	871	99307.7	5.58	13	2616	
*PtLOX17*	Potri.013G022100	1461474–1466287	862	98766.3	6.14	13	2589	
*PtLOX18*	Potri.014G018200	1725218–1731715	860	98729.1	6.6	14	2583	
*PtLOX19*	Potri.014G177200	14542431–14547953	860	98485.7	6.34	14	2583	
*PtLOX20*	Potri.017G046200	3854572–3860007	898	102778.8	5.73	17	2697	

A previous study has reported 64 plant lipoxygenase amino acid sequences in 16 plant species [55–58]. To gain insights into the evolutionary relationship among plant LOX proteins, we examined all of the sequences of these *LOX* genes and all of the whole genome sequence information that was available. A complete list of these *LOX* genes is provided in S3 Table.

### Evolutionary analysis of the Lipoxygenase genes

**Fig 1:**
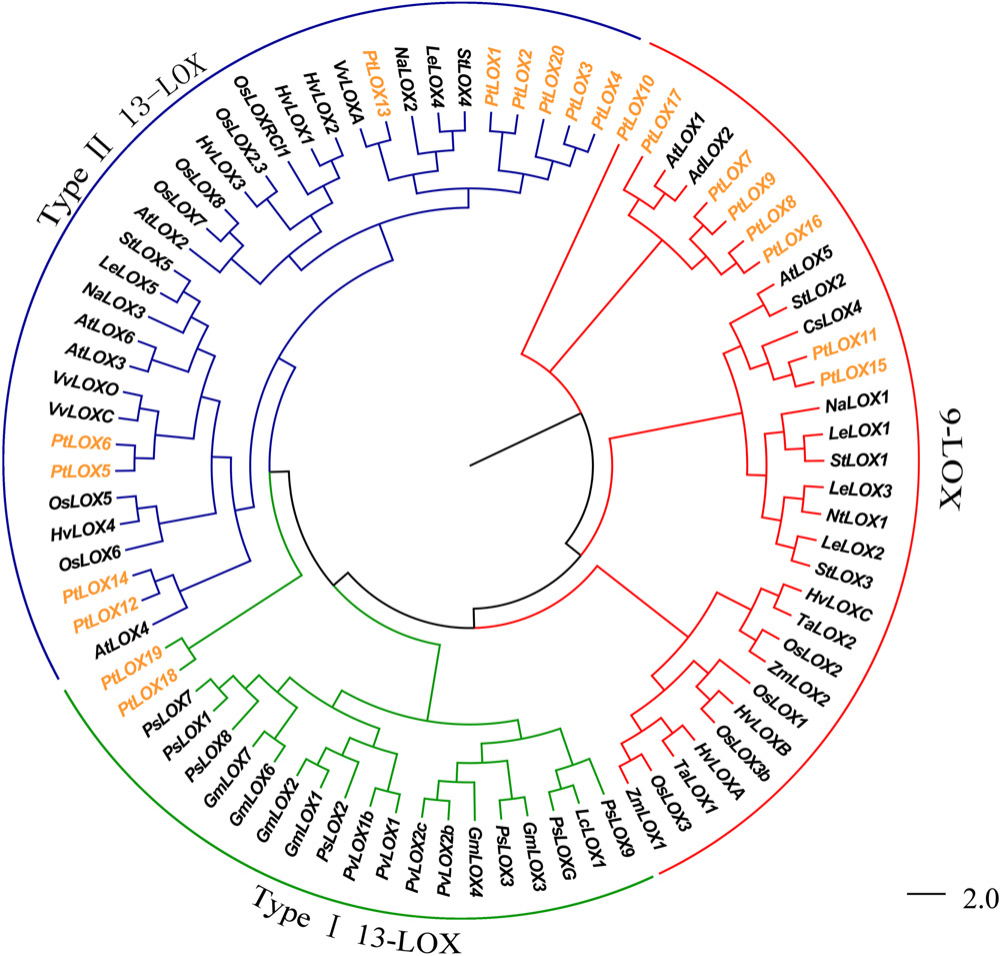
An analytical view of the *LOX* gene family. Bayesian estimate of phylogenetic relationships among the 84 members of LOX proteins from 17 species. Branch length is indicated by the scale bars. The tree was generated using MRBAYES program by Bayesian method and viewed in FigTree; Gene classes were indicated with different colors. Taxon labels are depicted in red for the 13-LOX clade, in blue for type II 13-LOX clade, in green for type I 13-LOX clade. Members of LOX protein from Populus were denoted in yellow.

The phylogenetic analyses of LOX proteins have been reported for some plants. They could help us to uncover the evolutionary relationship of the *LOX* genes. Phylogenetic trees were constructed using amino acid sequence alignments of conserved regions of all predicted proteins (S4 Table) in Bayesian inference (BI) and Neighbour-Joining (NJ) methods (Fig 1 and S2 Fig). The tree topologies produced by the two methods are largely consistent. The BI tree is shown in Fig 1 and discussed below. As mentioned above, plant lipoxygenases can be divided into 9-LOX or 13-LOX subfamilies, respectively [14]. 13-LOX proteins can be further categorized into two types with respect to their protein structure. Type I LOX proteins lack plastid targeting peptides and share sequence similarities of over 75%, whereas type II LOXs possess a plastid-targeting peptide and exhibit low levels of sequence similarity.

As shown in Fig 1, *PtLOX7-11* and *PtLOX15-17* were grouped with the characterized 9-LOX. The remaining LOXs were placed in the type I 13-LOXs group, with the exception of *PtLOX 1–4*, *PtLOX12-14* and *PtLOX20*, which belonged to the type II 13-LOX group. Upon closer inspection, we found that LOXs in 9-LOX subfamilies from monocotyledons and dicotyledons formed two exclusive clusters, respectively, which indicated that these *LOX* genes might have diverged after the monocotyledon/dicotyledon separation. In this subfamily, we found that *PtLOX* genes were closely aligned to *AtLOX1*, *AtLOX5*, *StLOX2* and *CsLOX4*. *AtLOX1* represents a regulatory point in the synthesis of jasmonic acid or other octadecanoids in response to pathogen attack [59]. *AtLOX5* had been proven to be involved in lateral root development and defense responses in Arabidopsis [60]. *CsLOX*4 is primarily induced upon mechanical wounding and its product exhibits fungicidal activity [61]; *StLOX2* was involved in herbivore-induced JA biosynthesis [62], which suggests that eight *PtLOXs* (*PtLOX7-11*, *PtLOX15-17*) genes may participate in the formation of JA. However, the detailed underlying mechanism of this function requires further research and confirmation.

Meanwhile, in the 13-LOX subfamily, the *LOX* genes exhibited an alternating distribution of monocots and eudicots, which suggested that an ancestral set of *LOX* genes may already have existed before the monocot-eudicot divergence. *PtLOX12* and *PtLOX14* were found to be closely related to *AtLOX4*. *AtLOX*4 plays an important role during seed development in Arabidopsis [63]. Genes with similar functions often are closely related and this has been confirmed in previous reports. Such a trend is also found in the *LOX* genes. For instances, an exclusive cluster that includes *LeLOX4* [64], *StLOX4* [65], *NaLOX2* [66], and *VvLOXA* [55], all of which play important roles in protecting plants from biotic attack by producing defensive compounds. So we conjectured that *PtLOX1-4*, *PtLOX13*, and *PtLOX20* could serve a similar function. Other members in this subfamily, *LeLOX5* [67], *StLOX5* [67], *NaLOX3* [68], and *VvLOXO* [55] can induce plant defense mechanisms that are effective against pathogens. We can clearly see that *PtLOX18* and *PtLOX19* have a far-reaching genetic relationship with other members in this subfamily. It is possible that these genes have specialized roles in poplar.

### Phylogenetic and structural analysis of *PtLOX* genes

**Fig 2:**
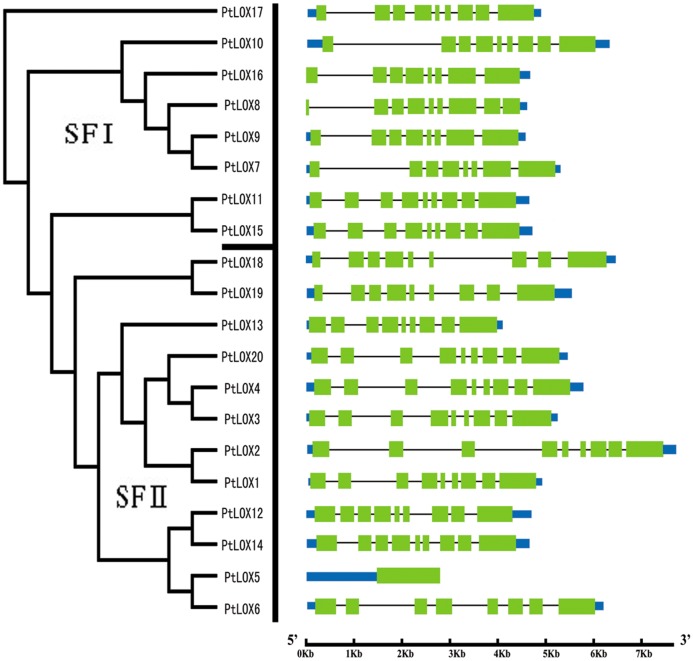
Phylogenetic relationship and gene structure of Populus *LOX* genes. The phylogenetic tree was constructed by the Neighbor-Joining (NJ) method with 1,000 bootstrap replicates. The two PtLOX phylogenetic subfamilies were designated as I–II. Exons and introns are represented by green boxes and gray lines, respectively. The sizes of exons and introns are proportional to their sequence lengths.

To gain further insight into the structural diversity of poplar *LOX* genes, we constructed a separate phylogenetic tree using only the poplar LOX protein sequences and compared the exon/intron organization in the coding sequence of each poplar *LOX* gene (Fig 2). The *PtLOX* genes were divided into two subfamilies; class I contains eight members, which are all 9-LOX genes, whereas class II consists of 12 members, all of which are 13-LOX genes.

In our study, most members of the poplar *LOX* gene family were found to share similar exon/intron structures either in terms of intron numbers or exon length. As shown in Fig 2, most of the *LOX* genes harbor eight to nine introns in each of their open reading frames. Remarkably, only *PtLOX5* lacks an intron. The emergence of a gene with no introns might result from a recent intron acquisition [69].

**Fig 3:**
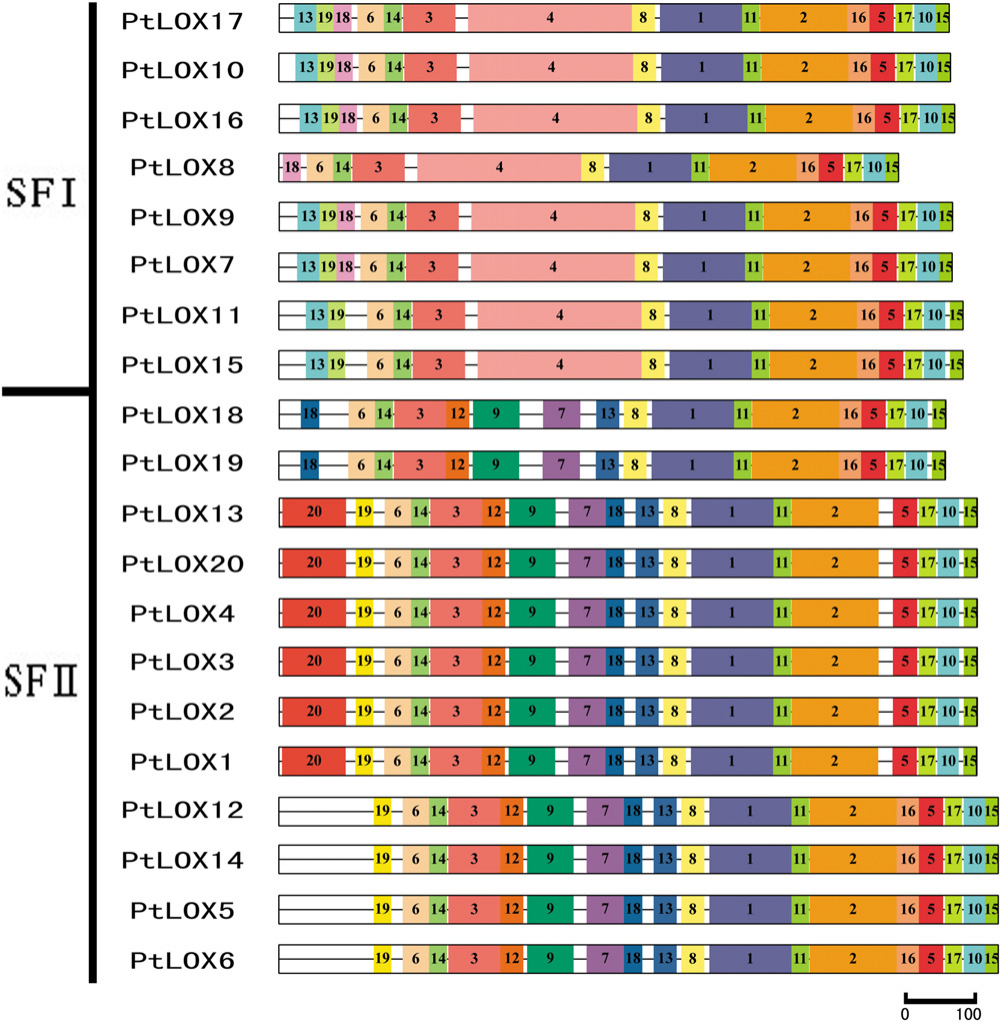
Schematic representation of the conserved motifs in Populus LOX proteins elucidated using publicly available information. Each colored box represents a motif in the protein, with the motif number indicated in the middle of the box. The length of the protein and motif can be estimated using the scale at the bottom.

**Fig 4:**
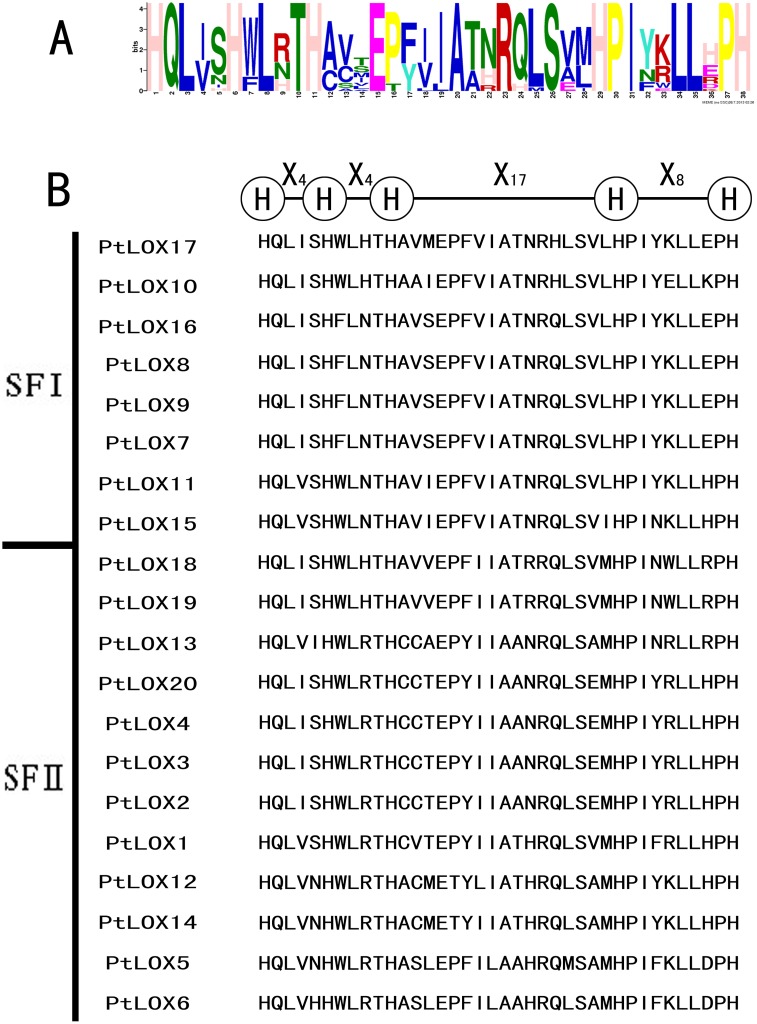
A 38-residue motif among Populus LOX sequences. A. The logo was created with 20 Populus LOX sequences. The overall height of each stack indicates the sequence conservation at that position and the height of each residue letter indicates the relative frequency of the corresponding amino acid residue. Below the logo is the consensus sequence. The conserved histidines (H) are marked with boldface letters. B. Sequence alignments of 38-residue motifs in Populus LOX members.

To reveal conserved motifs shared among related proteins within the family, we predicted and annotated the putative motifs. Ultimately, 20 distinct motifs were identified in the poplar LOX proteins (Fig 3 and S5 Table). As expected, most of the closely related members had common motif compositions, suggesting functional similarities among LOX proteins within the same subfamily. Based on the results, we determined that 14 of the motifs (motif 1–3, 5–6, 8, 10–11, and 13–19) are shared among all of the LOX proteins. A region rich in histamines residues was previously observed in the primary structure of soybean LOX isozymes, and further investigation has shown that His499, His504, His690, Asn694, and Ile839 are essential for the binding of iron in the C-terminal amino acid. A conservative region was also identified in 20 PtLOX members. Motif 1, which includes the representative 38 amino acid residues [24, 50, 51, 70], was in accordance with the structure His-(X)4-His-(X)4-His-(X)17-His-(X)8-His identified in a previous study; this motif is highly conserved (Fig 4). This motif plays an important role in enzyme stability and substituting any of the residues in this motif alters the activity of this enzyme.

Notably, some specific motifs were present in LOXs from specific subfamilies. For instance, motif 4 was only detected in subfamily I *LOX* genes. Subfamily II LOXs contain the common motifs as well as other motifs (7, 9, 12, 13, and 20). Using Pfam[71], we determined that most of these motifs were LOX [50, 51]. However, whether the two novel motifs (19, 20) confer unique functional roles to LOXs remains to be investigated. In addition, we used four different kinds of software to predict the subcellular localization of these LOXs (S6 Table). Within the motif 20, proteins with extended N-termini (Fig 4) were predicted to be chloroplast-localized (S6 Table). The N-terminal regions were less conserved in both groups, harboring putative transit peptides for sub-cellular targeting.

In any case, the presence of conserved motifs in the LOX proteins from the same class provides additional support to the results of the phylogenetic analyses. On the other hand, the divergence in motif composition among different classes may indicate that the classes are functionally diversified.

### Chromosomal location and gene duplication of poplar *LOX* genes

**Fig 5:**
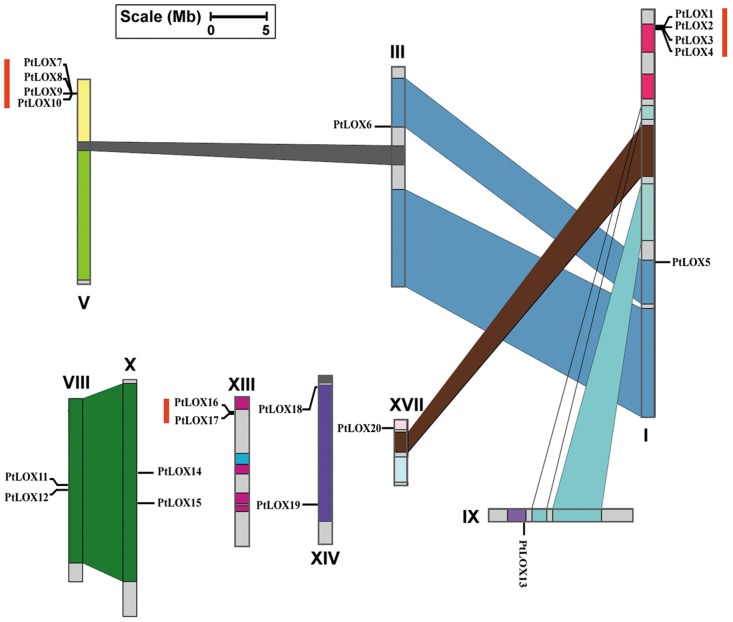
Chromosomal locations of the Populus *LOX* genes. Twenty genes were mapped to 9 of 19 Linkage Groups (LG). The schematic representation of genome-wide chromosome organization that arose from the whole-genome duplication event in Populus was obtained from Tuskan et al. Segmental duplicated homologous regions are shown with the same color. The duplication blocks containing *LOX* genes are connected with lines. Eight tandemly duplicated genes within 60 kb (shown in the red box) were organized into three clusters. Scale at the bottom represents a 5-Mb chromosomal distance.

Chromosomal locations were determined based on the microsatellite-based genetic map for *P*. *trichocarpa × P*. *deltoides*. As shown in Fig 5, *PtLOX* genes are present on 9 of the 19 chromosomes (Fig 5; S7 Table). The distribution of poplar *LOX* genes among the chromosomes appears to be uneven; chr. 3, 8, 9, 10, 13, 14, and 17 harbor one or two *LOX* genes, while relative high densities of *LOX* genes were observed in some locations on chr. 1 and chr. 5. The number of *LOX* genes on these chromosomes is 5 and 4, respectively.

A genome-wide duplication event occurred approximately 65 million years ago (Mya)[36]. Identification of homeologous chromosome segments resulting from whole-genome duplication events has been reported. Considering the complexity of LOX regulation, evolutionary systems biology studies are needed to explore the reasons for and implications of diversification. Based on the database from the VISTA browser, we found that three duplicate pairs (*PtLOX5/6*, *PtLOX11/15*, and *PtLOX12/14*) are each located in segmental duplication regions. These results suggest that the three *PtLOX* gene pairs (30% of the genes) are associated with the recent salicoid duplication event [36]. Additionally, comparisons of the 60-kb flanking genomic regions of the paralogous genes *PtLOX1/2/3/4*, *PtLOX7/8/9/10* and *PtLOX16/17* showed that these duplicate gene pairs were formed by a tandem duplication event. These ten *PtLOX* genes (50% of total) are represented in three distinct tandem duplicate gene clusters, with two clusters containing four tandem genes and one cluster containing two tandem genes. In addition, there are still four *PtLOX* genes that lack the corresponding duplicates, suggesting that dynamic changes may have occurred following gene duplication, which resulted in the loss of some genes.

Previous studies have shown that approximately 14,000 of the 45,000 (~32%) predicted genes are retained in duplicated pairs resulting from the salicoid duplication event [72]. In the current study, the retention rate for *PtLOX* family genes was found to be 30%. With such a high proportion of replication, 50% of the genes are represented in tandem clusters; this proportion is much higher than that of chromosome-level segmental duplication events. These observations suggest that the *PtLOX* family consists of genes originating primarily from high tandem duplication events and secondarily from low segmental duplication events. The amplification of *LOX* gene may due to environmental changes adaption [73].

To analyze the positive or negative selection of specific amino acid sites within the full-length sequences of the LOX proteins in the different LOX groups, we calculated the substitution rate ratios of nonsynonymous (Ka) versus synonymous (Ks) mutations [74]. The ratio of nonsynonymous versus synonymous substitutions (Ka/Ks) is an indicator of the history of selection acting on a gene or gene region. Ratios significantly <0.5 suggest purifying selection for both duplicates. A summary of Ka/Ks values for seven LOX paralogous pairs is shown in Table 2. The results suggest that all gene pairs except PtLOX18/19 have evolved mainly under the influence of purifying selection. In this study, the Ka/Ks ratio of seven putative paralogous gene pairs identified were calculated to reveal the divergence fate after duplication of poplar LOX and its closely related genes. Most Ka/Ks ratios (87.5%) of all paralogous pairs were no larger than 0.4 (Table 2). We therefore conclude that the poplar *LOX* gene family has undergone great purifying selection pressure and the *LOX* genes are slowly evolving at the protein level [75]. In addition, based on the divergence rate of 9.1×10^-9^ synonymous mutations per synonymous site year proposed for poplar[76], duplications of the paralogous gene pairs were estimated to occur between 0.80 and 30.87 Mya (Table 2).

**Table 2:** Divergence between paralogous LOX gene pairs in Populus.

Paralogous pairs	Ka	Ks	Ka/Ks	Duplication date (MY)
*PtLOX1–PtLOX2*	0.1418	0.5618	0.2524	30.86813187
*PtLOX3–PtLOX4*	0.0029	0.0146	0.2005	0.802197802
*PtLOX5–PtLOX6*	0.0732	0.0288	0.254	1.582417582
*PtLOX7–PtLOX9*	0.0072	0.0181	0.3982	0.994505495
*PtLOX11–PtLOX15*	0.0425	0.1884	0.2253	10.35164835
*PtLOX12–PtLOX14*	0.0578	0.2396	0.2412	13.16483516
*PtLOX18–PtLOX19*	0.0224	0.0327	0.6847	1.796703297

### Expression profiling of poplar *LOX* genes

**Fig 6:**
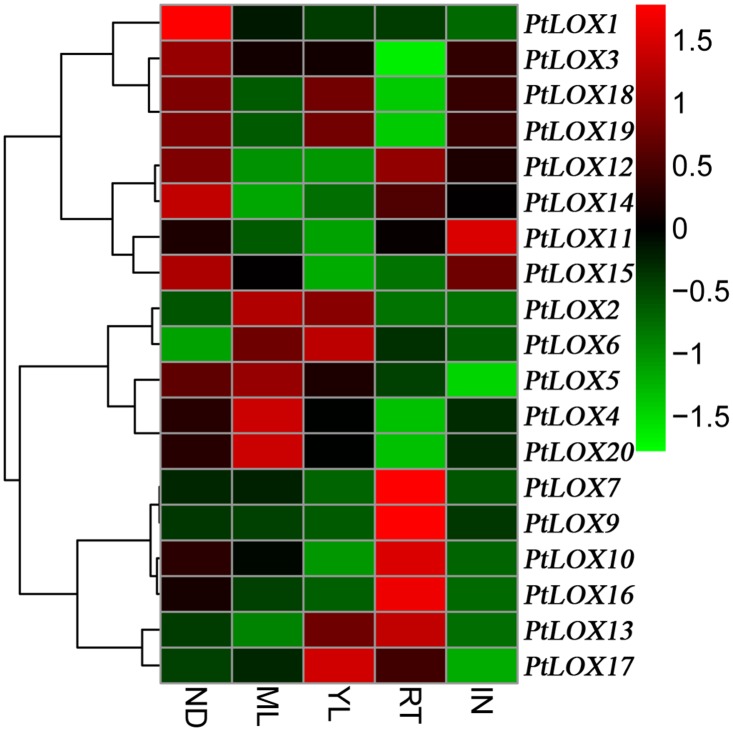
Expression profiles of Populus *LOX* genes across different tissues. Background-corrected expression intensities were log-transformed and visualized as heatmaps (see Materials and Methods). YL, young leaves; ML, mature leaves; RT, roots; IN, internodes; ND, nodes.

In order to understand the expression patterns of *LOX* genes, we data mined the publicly available microarray data. Firstly, we analyzed the expression of *LOX* genes in various tissues/organs. The microarray data set was obtained from PopGenIE [39]. Distinct expression profiles were identified for a total of 19 *LOX* genes (Fig 6 and S8 Table), whereas no corresponding probe was available for *PtLOX8*. Under normal growing conditions, the majority of *LOX* genes were expressed by only one or two tissues. There is no gene highly expressed in all tested tissues. For example, *PtLOX7*, *-9*, *-10*, *-13*, and *-16* exhibited high levels of expression in roots. *PtLOX2*, *4–6*, and *-20* were present at high levels in the mature leaves, while *PtLOX1* and *14–15* were mainly expressed in nodes. Thus, *LOX* genes showed divergent mRNA expression patterns across different tissues.

**Fig 7:**
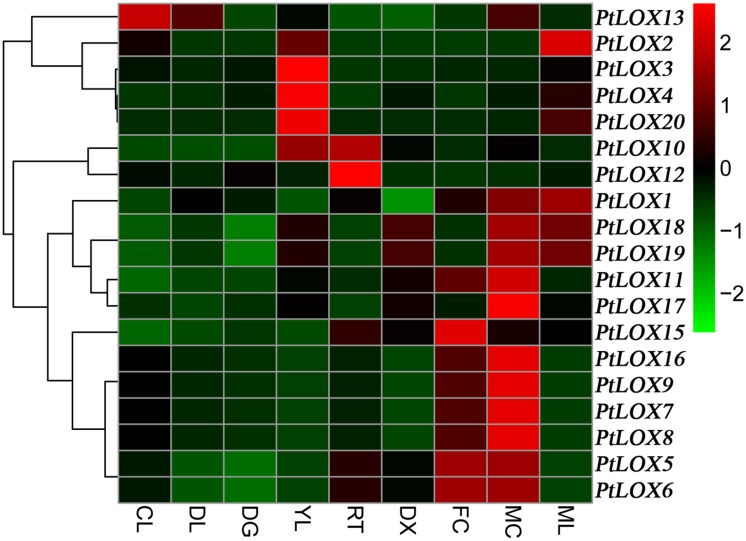
Hierarchical clustering of expression profiles of Populus *LOX* genes in different tissues. Background-corrected expression intensities were log-transformed and visualized as heatmaps (see Materials and Methods). YL, young leaves; ML, mature leaves; RT, roots; FC, female catkins; MC, male catkins; XL, xylem. CL, continuous lightgrown seedling; DL, etiolated dark-grown seedling transferred to light for 3 h; DG, dark-grown seedlings, Etiolated seedling.

To gain further insights into the expression profiles of *LOX* genes, we performed a comprehensive expression analysis using some of the publicly available microarray data for Populus. Nineteen *LOX* genes were included on GSE13990, and only one gene (*PtLOX14*) was absent. Microarray data indicate that almost all *LOX* genes have minimal changes to each other when present in seedlings under specific conditions (CL, continuous lightgrown seedling; EL, etiolated dark-grown seedling transferred to light for 3 h; DS, dark-grown seedlings, Etiolated seedling) (Fig 7 and S9 Table). It appears that the duration of sunshine played little or no role in the distribution of *LOX* genes. The largest fraction of *LOX* genes was preferentially expressed in mature leaves (ML; 10/19), and male catkins (MC; 14/19). A relatively large fraction of *LOX* genes was expressed in female catkins (FC; 8/19), young leaves (YL; 10/19) and differentiating xylem (DX; 7/19). Higher expression levels of *LOX* genes in these parts might contribute to many developmental and biological processes. According to the expression patterns of Populus genes and the functions of their Arabidopsis orthologs, we are able to hypothesize the possible functions of these genes in Populus. For example, *PtLOX15* is orthologous to *AtLOX5*[60], showed high expression levels in root. *AtLOX5* has strong levels of biological activity and is likely to help regulate lateral root development. Therefore, *PtLOX15* might have a function that is similar to *AtLOX5* in the regulation of Populus root development. However, some *LOX* genes appear not to follow this trend. For example, *PtLOX5* and *PtLOX6* displayed low levels of expression in seed. However, the ortholog of *PtLOX5* and *PtLOX6* in Arabidopsis is *AtLOX3*[77], which plays an important role during seed development. This discrepancy in *LOX* gene regulation may suggest that LOXs adopt different physiological functions in these two plant species.

**Fig 8:**
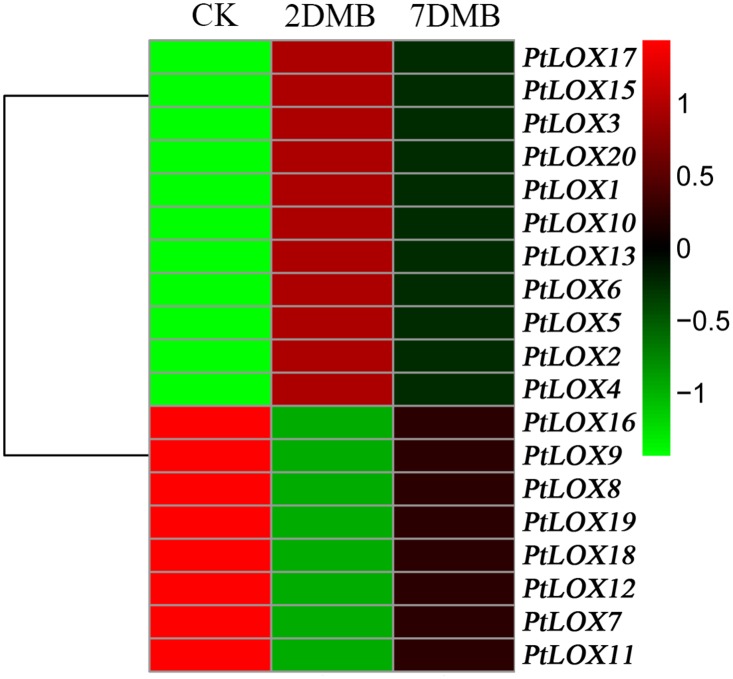
Differential expression of Populus *LOX* genes under different stress treatments. Expression is indicated as fold-change of experimental treatments relative to control samples and visualized in heatmaps (see Materials and Methods). Green represents low levels and red indicates high levels of transcript abundance. Microarray data under the series accession number GSE23726 were obtained from the NCBI GEO database. CK: 0 days after inoculation with Marssonina brunnea; 2DMB: 2 days after inoculation with Marssonina brunnea; 7DMB: 7 days after inoculation with Marssonina brunnea.

**Fig 9:**
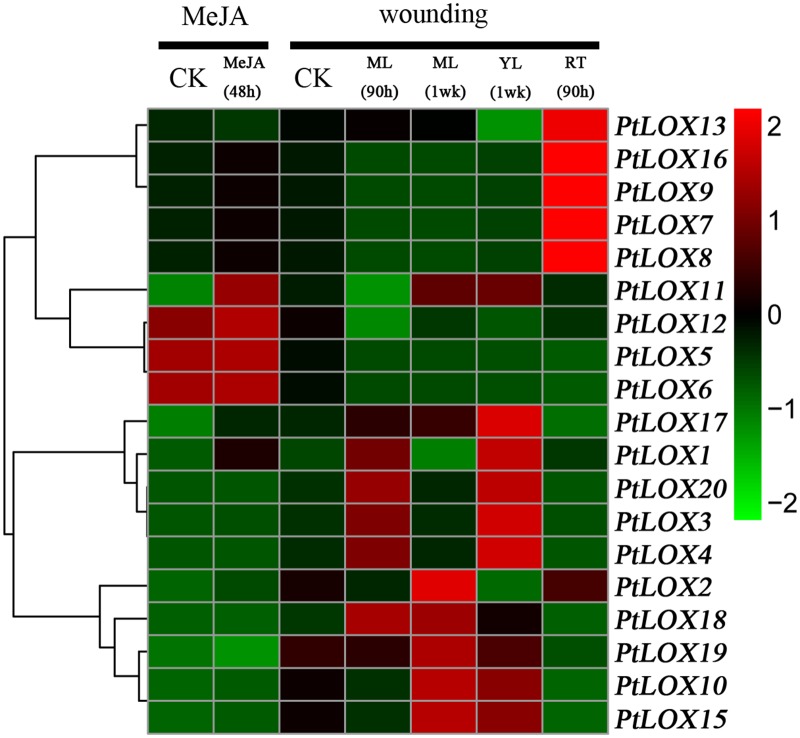
Differential expression of Populus *LOX* genes under different stress treatments. Expression is indicated as fold-change of experimental treatments relative to control samples and visualized in heatmaps (see Materials and Methods). Green represents low levels and red indicates high levels of transcript abundance. Heatmap showing hierarchical clustering of Populus *LOX* genes under leaf wounding and methyl jasmonate treatment. Microarray data under the series accession number GSE16786 were obtained from the NCBI GEO database. Tissues analyzed included: YL, young leaves; ML, mature leaves; RT, root tips; C, suspension cell cultures. Stress treatments included: MeJA, Methyl Jasmonate elicitation; wounding, sampled either one week or 90 hours after wounding.

The quality of poplar is strongly affected by environmental cues during the development of the plant. Therefore, we examined the expression patterns of the Populus *LOX* genes under different stress conditions including infection with *Marssonina brunnea* (Fig 8 and S10 Table), Methyl Jasmonate (MeJA) treatment, and mechanical wounding (Fig 9 and S11 Table). The gene expression of most *LOX* genes is induced or suppressed under these biotic and abiotic stresses. With *Marssonina brunnea* infections (Fig 8), the expression levels of some genes, such as *PtLOX1-6* -10, -13, -15, -17 and -20, initially increased and then decreased. Reversely, the expression levels of *PtLOX7-9*, *11–12*, *-16*, *-18* and *-19* increased after they decreased. In response to MeJA treatments of cells in culture (Fig 9), *LOX* genes including *PtLOX3*, *-4*, *-10*, *-13* and *-18* were unresponsive to this stress. *PtLOX15*, *-19* was shown to be slightly down-regulated whereas the transcripts of 12 genes (*PtLOX1-2*, *5–9*, *11–12*, *16–17*, and *-20*) showed up-regulation. Mechanical wounding stress caused commonly down-regulation of two genes (*PtLOX5* and *6*) at 90 h and/or one week after wounding in young leaves, expanding leaves and root tips. In addition, a subset of genes showed up- or down-regulation only under mechanical wounding stress with root. Similarly, transcripts of a considerable proportion of genes were either enhanced or repressed following mechanical wounding in young leaves or mature leaf, respectively(Fig 9). Duplicated genes may have different evolutionary fates, which are indicated by the divergence of their expression patterns.

Duplicated genes may have different evolutionary fates, which are indicated by the divergence of their expression patterns. Of the seven paralogous pairs of *LOX* genes, one gene had no corresponding database. For the remaining six paralogous pairs of *LOX* genes, the expression analysis of two pairs of paralogs (*PtLOX5 and PtLOX6*, *PtLOX7* and *PtLOX9*) showed that they had similar expression profiles. On the contrary, one gene pair (*PtLOX11* and *PtLOX15*) shared disparate expression patterns (Figs 8 and 9), which indicated that substantial neofunctionalization may have occurred during the evolution of one pair of genes. Beyond that, four genes (*PtLOX1-4*) derived from tandem duplication shared partial identical expression patterns, which suggests the functional diversification of duplicated genes [78, 79]. Such a process could increase the adaptability of duplicated genes to environmental changes, and thus improve product quality and military benefits.

### Examination of *LOX* gene expression by qRT-PCR

**Fig 10:**
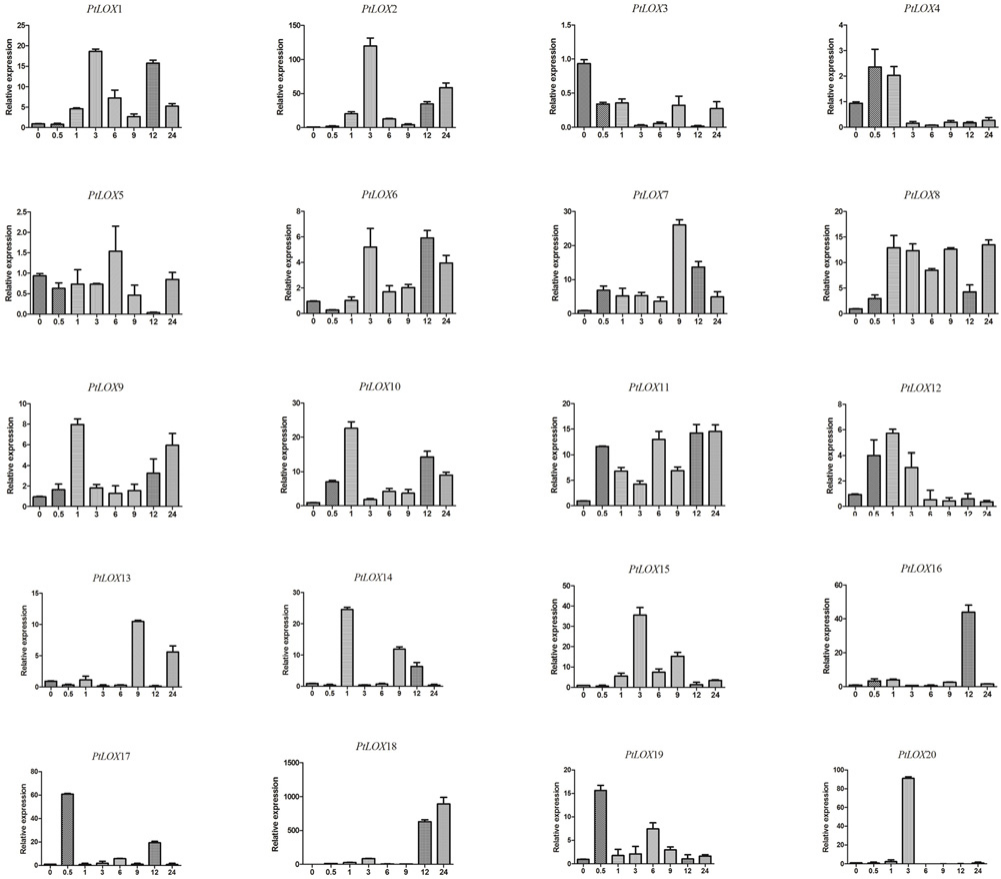
Expression analysis of *PtLOX* genes after MeJA induction. Sampling occurred 0, 0.5, 1, 3, 6, 9 12 and 24 h after treatment, and the relative expression levels were analyzed. Untreated sample expression levels = 1. X-axes represent time points after MeJA treatment. Y-axes represent relative gene expression values normalized to reference gene *UBQ10*. Bars indicate standard deviations (SD) from three biological replicates.

Transcript levels of the poplar *LOX* genes response to MeJA treatments were investigated using qRT-PCR. As shown in Fig 10A, one gene, *PtLOX3*, had low expression levels throughout the period, while the expression level of two other genes (*PtLOX4* and *-5*) had no significant difference during each period. In addition to the three genes, 17 out of the 20 genes (*PtLOX1-2* and *-6-20*) (Fig 10) were upregulated in the leaves of MeJA-treated poplar, and one gene (*PtLOX18*) (Fig 10) showed a significant level of upregulation after 24 h. *PtLOX2* expression was upregulated within 3 h, and then its level of expression level decreased after MeJA treatment; however, the expression level of this gene showed an increasing trend after 9 h; the expression pattern of *PtLOX9* (Fig 10A) and *PtLOX13* (Fig 10) was similar to this. The expression of *PtLOX1* and *PtLOX6* (Fig 10) were also increased by MeJA treatment, reaching their maximum levels of expression after 3 h; after this time point, levels of expression was maintained above the basal levels for up to 24 h. Transcripts of *PtLOX14*, *-16*, *-17*, and *-20* (Fig 10) were strongly induced by MeJA treatment, which peaked at some point in time, and this expression declined over time. Besides, the expression of *PtLOX7*, *PtLOX8*, *PtLOX11* and *PtLOX15* (Fig 10) were induced by the MeJA treatment and showed no significant changes in transcript levels during each time point after treatment. However, unlike those genes, the expression of *PtLOX12* (Fig 10) reached a peak value at 3 h post-treatment. With increasing time, expression levels were lower than baseline levels at 6 h after treatment.

By comparing the duplicated pairs of *LOX* genes, we observed that *PtLOX11/-15* and *PtLOX16/-17* (Fig 10) showed that they have similar expression profiles. This finding indicated that the responses of paralogs to stress conditions did not undergo a significant level of divergence along with the evolution of each gene after duplication. By contrast, other gene pairs *PtLOX1/-2/-3/-4*, *PtLOX5/-6* and *PtLOX12/-14* (Fig 10) shared disparate expression patterns. This is a sign of differentiation and may aid in the selection of appropriate candidate genes for further research.

## Conclusions

Considerable effort has been made to characterize LOXs in many plants, including cucumber, rice, and Arabidopsis, amongst others, but such efforts have not previously been directed towards poplar. This study identified the suites of *LOX* genes in the poplar genome including their phylogeny, chromosomal location, gene structure, conserved motifs, and expression profiling. Finally, a total of 20 full-length *LOX* genes in poplar genome were identified. We searched the NCBI database for candidate poplar *LOX* genes and compared their protein sequences with the sequences of 64 *LOX* genes from 16 other plant species using phylogenetic analysis. All of the *LOX* genes in the draft poplar genome were verified and classified into two classes. Poplar *LOX* genes were clustered into two distinct subfamilies based on phylogenetic analysis. In each subfamily, the exon/intron structure and motif compositions of the LOXs were highly similar, which is indicative of their functional conservation. All PtLOX proteins contain a conventional His-(X)_4_-His-(X)_4_-His-(X)_17_-His-(X)_8_-His motif, indicating that these conserved motifs may play critical roles in family-specific functions. Chromosome distribution analysis revealed that the putative *LOX* genes are dispersed in 9 of 19 chromosomes. A high proportion of *LOX* genes are distributed preferentially at duplicated blocks, suggesting that tandem duplications contributed significantly to the expansion of this gene family. Expression analysis revealed that these *PtLOX* genes are expressed at higher levels in leaf tissues than in other tissues. *LOXs* play an important role in plant responses to multiple stresses. Furthermore, expression profiles by quantitative real-time PCR revealed that the identified PtLOXs have different patterns of expression in response to stresses. This result may reflect the involvement of these genes in specific stress-related functions.

This comprehensive analysis represents an important starting point for future efforts to elucidate the functional roles of all LOX proteins in poplar. The new information presented in this study may help in the selection of appropriate candidate genes for further functional characterization of basic aspects of the functional contexts of LOX proteins.

## Supporting Information

S1 Fig Standard curve analysis of each primer pairs. A: *UBQ10*; B:*PtLOX1*; C:*PtLOX2*; D:*PtLOX3*; E:*PtLOX4*; F: *PtLOX5*; G:*PtLOX6*; H: *PtLOX7*; I: *PtLOX 8*; J: *PtLOX9*; K: *PtLOX10*; L: *PtLOX11*; M: *PtLOX12*; N: *PtLOX13*; O: *PtLOX 14*; P: *PtLOX15*; Q: *PtLOX16*; R: *PtLOX17*; S: *PtLOX18*; T: *PtLOX19*; U: *PtLOX20*.
(TIF)

S2 Fig An analytical view of the LOX gene family. Protein Neighbor-Joining tree: The unrooted tree, constructed using ClustalX 2.0, summarizes the evolutionary relationship among the 84 members of LOX proteins from 17 species. The Neighbor-Joining tree was constructed using aligned full-length amino acid sequences. The proteins are named according to their gene names (see Table 1 and S3 Table). The tree shows the two major phylogenetic classes (named 9-LOX, 13-LOX and marked with different colors) with high predictive value. The blue circle surrounds.
(TIF)

S1 Table List of primer sequences of 20 *LOX* genes used for qRT-PCR analysis. (XLS)

S2 Table Coding region nucleotide (upper portion of matrix) and amino acid (bottom portion of matrix) sequence pairwise comparisons (% similarity) between poplar sucrose synthase genes. (XLSX)

S3 Table Plant lipoxygenase sequences used for phylogenetic analysis. (XLSX)

S4 Table Multiple sequence alignment of conserved regions of 84 LOX protein sequences. (XLSX)

S5 Table Sequence logos for the conserved motifs of Populus LOX proteins. Conserved motifs and the sequence logos were generated using the MEME search tool.
(XLS)

S6 Table Subcellular localization of Populus LOX proteins. (XLSX)

S7 Table A complete list of 20 LOX gene sequences identified in the present study. Genomic sequences were obtained from Phytozome (http://www.phytozome.net/).
(XLS)

S8 Table Probes and microarray data extracted from PopGENIE. YL, young leaves; ML, mature leaves; RT, roots; IN, internodes; ND, nodes.
(XLSX)

S9 Table Probes and microarray data used for analysis of poplar LOX genes. YL, young leaves; ML, mature leaves; RT, roots; FC, female catkins; MC, male catkins; XL, xylem. CL, continuous lightgrown seedling, EL, etiolated dark-grown seedling transferred to light for 3 h, DS, dark-grown seedlings, Etiolated seedling.
(CSV)

S10 Table Probes and microarray data used for the analysis of poplar *LOX* genes. CK: 0 days after inoculation with *Marssonina brunnea*; 2DMB: 2 days after inoculation with *Marssonina brunnea*; 7DMB: 7 days after inoculation with *Marssonina brunnea*.
(CSV)

S11 Table Probes and microarray data used for analysis of poplar *LOX* genes. CK: cell culture, control; MeJA, methyl jasmonate elicitation; wounding, sampled at 90 hours (W90h) or 1 week (W1W) after wounding.
(CSV)
