# Supplementary figures and images for: The Lipoxygenase Gene Family in Poplar: Identification, Classification, and Expression in Response to MeJA Treatment
Source: PLoS One. 2015 Apr 30;10(4):e0125526. doi: 10.1371/journal.pone.0125526 (PMC4415952; doi:10.1371/journal.pone.0125526)

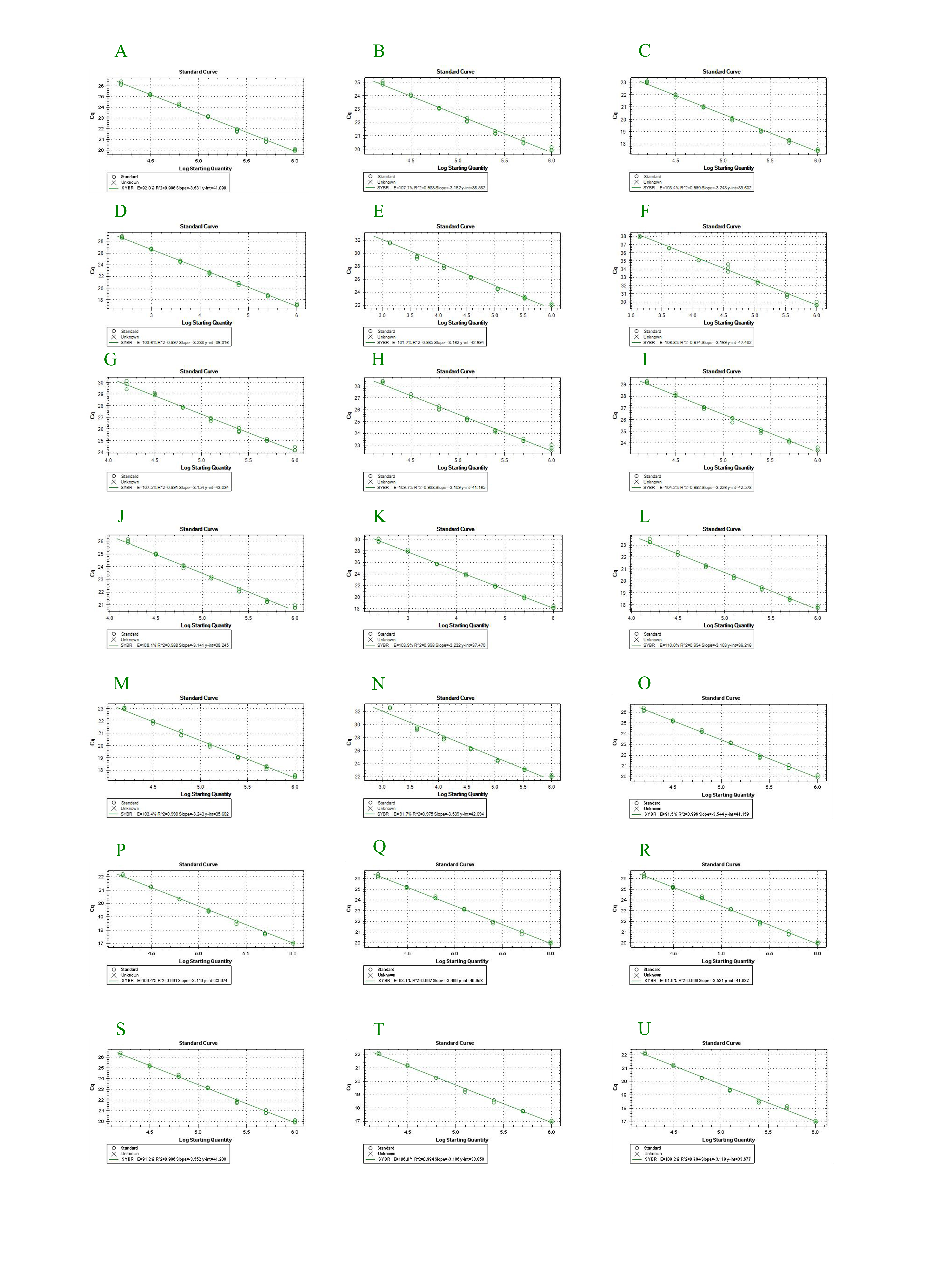

Supplement: S1 Fig — A: UBQ10; B:PtLOX1; C:PtLOX2; D:PtLOX3; E:PtLOX4; F: PtLOX5; G:PtLOX6; H: PtLOX7; I: PtLOX 8; J: PtLOX9; K: PtLOX10; L: PtLOX11; M: PtLOX12; N: PtLOX13; O: PtLOX 14; P: PtLOX15; Q: PtLOX16; R: PtLOX17; S: PtLOX18; T: PtLOX19; U: PtLOX20. (TIF) [file pone.0125526.s001.tif]

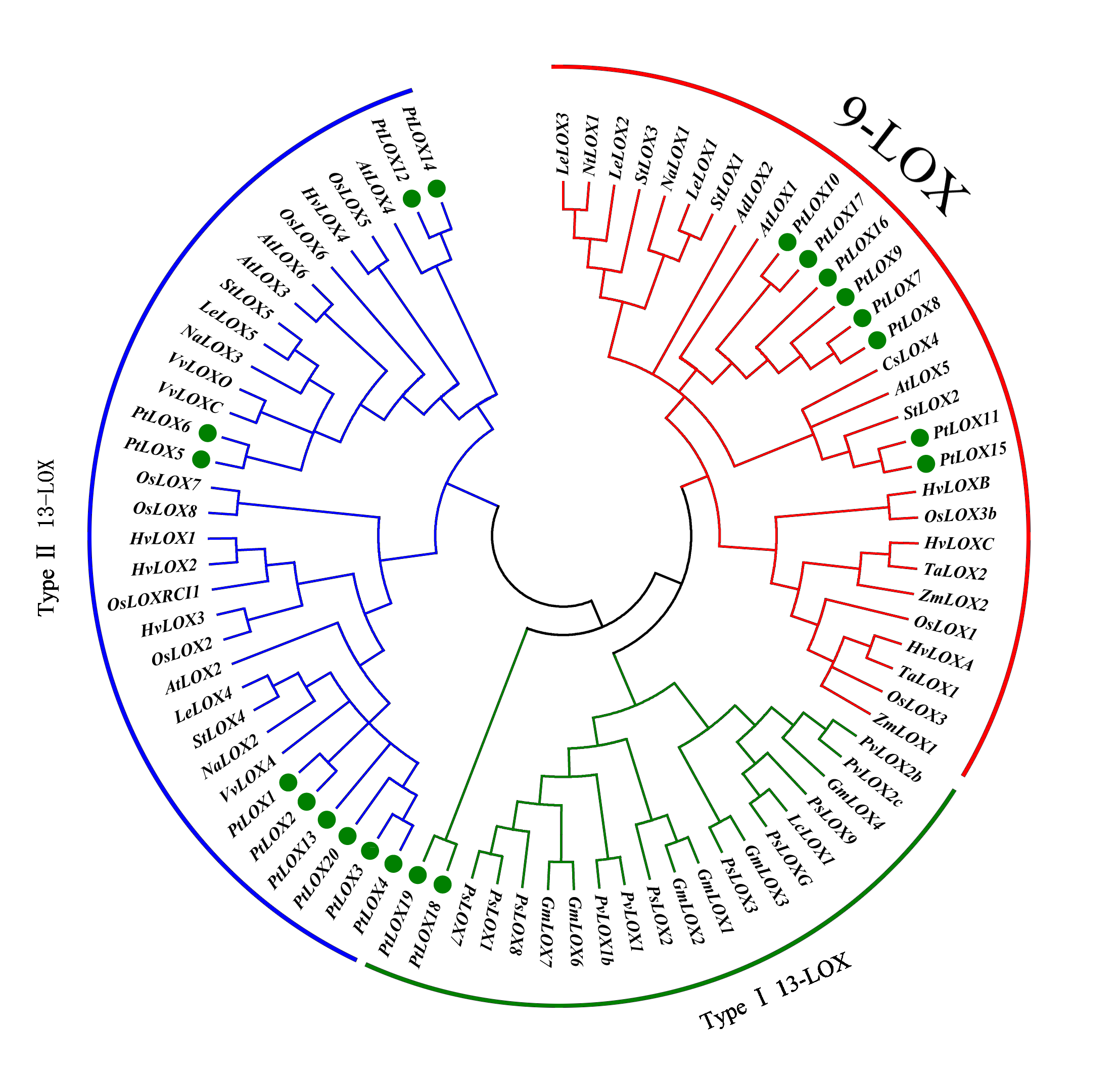

Supplement: S2 Fig — Protein Neighbor-Joining tree: The unrooted tree, constructed using ClustalX 2.0, summarizes the evolutionary relationship among the 84 members of LOX proteins from 17 species. The Neighbor-Joining tree was constructed using aligned full-length amino acid sequences. The proteins are named according to their gene names (see Table 1 and S3 Table). The tree shows the two major phylogenetic classes (named 9-LOX, 13-LOX and marked with different colors) with high predictive value. The blue circle surrounds. (TIF) [file pone.0125526.s002.tif]
